# Ploidy mosaicism and allele-specific gene expression differences in the allopolyploid *Squalius alburnoides*

**DOI:** 10.1186/1471-2156-12-101

**Published:** 2011-12-05

**Authors:** Isa Matos, Élio Sucena, Miguel P Machado, Rui Gardner, Ângela Inácio, Manfred Schartl, Maria M Coelho

**Affiliations:** 1 Centro de Biologia Ambiental, Departamento de Biologia Animal, Faculdade de Ciências da Universidade de Lisboa, Campo Grande, Lisbon, 1749-016, Portugal; 2 Physiologische Chemie I, Biozentrum, University of Würzburg, Am Hubland Würzburg, 97074, Germany; 3 Instituto Gulbenkian de Ciência, Rua da Quinta Grande, Oeiras, 2780-156, Portugal; 4 Departamento de Biologia Animal, Faculdade de Ciências da Universidade de Lisboa, Campo Grande, Lisbon, 1749-016, Portugal

## Abstract

**Background:**

*Squalius alburnoides *is an Iberian cyprinid fish resulting from an interspecific hybridisation between *Squalius pyrenaicus *females (P genome) and males of an unknown *Anaecypris hispanica-*like species (A genome). *S. alburnoides *is an allopolyploid hybridogenetic complex, which makes it a likely candidate for ploidy mosaicism occurrence, and is also an interesting model to address questions about gene expression regulation and genomic interactions. Indeed, it was previously suggested that in *S. alburnoides *triploids (PAA composition) silencing of one of the three alleles (mainly of the P allele) occurs. However, not a whole haplome is inactivated but a more or less random inactivation of alleles varying between individuals and even between organs of the same fish was seen.

In this work we intended to correlate expression differences between individuals and/or between organs to the occurrence of mosaicism, evaluating if mosaics could explain previous observations and its impact on the assessment of gene expression patterns.

**Results:**

To achieve our goal, we developed flow cytometry and cell sorting protocols for this system generating more homogenous cellular and transcriptional samples. With this set-up we detected 10% ploidy mosaicism within the *S. alburnoides *complex, and determined the allelic expression profiles of ubiquitously expressed genes (*rpl8*; *gapdh *and *β-actin*) in cells from liver and kidney of mosaic and non-mosaic individuals coming from different rivers over a wide geographic range.

**Conclusions:**

Ploidy mosaicism occurs sporadically within the *S. alburnoides *complex, but in a frequency significantly higher than reported for other organisms. Moreover, we could exclude the influence of this phenomenon on the detection of variable allelic expression profiles of ubiquitously expressed genes (*rpl8*; *gapdh *and *β-actin*) in cells from liver and kidney of triploid individuals. Finally, we determined that the expression patterns previously detected only in a narrow geographic range is not a local restricted phenomenon but is pervasive in rivers where *S. pyrenaicus *is sympatric with *S. alburnoides*.

We discuss mechanisms that could lead to the formation of mosaic *S. alburnoides *and hypothesise about a relaxation of the mechanisms that impose a tight control over mitosis and ploidy control in mixoploids.

## Background

The chromosome theory of heredity rests on the consistency and stability of chromosome number and composition [1]. This consistency and stability is achieved by the existence of extremely precise and tightly controlled mechanisms of chromosome replication and segregation during cell divisions [2]. However, genetic information and the way it is inherited are not so invariant and rigorous as previously thought [3]. Experimental findings in reproductive genetics have shown that basic processes such as mitosis, meiosis/gametogenesis, fertilization and embryogenesis are often imprecise and present some level of plasticity [4]. It is through this mechanistic plasticity and the ability of organisms to cope with seemingly low frequencies of genetic aberrations that hybridization and polyploidy emerge as naturally occurring phenomena. In this light, allopolyploids, like the cyprinid fish *Squalius alburnoides*, constitute a paradigmatic example of successful escapers from the canonical rules of reproductive biology and heredity [5-9].

The *Squalius alburnoides *complex is endemic from the Iberian Peninsula. It resulted from interspecific hybridisation between females of *Squalius pyrenaicus *(P genome) and males of an unknown species related to *Anaecypris hispanica *(A genome) [reviewed in [10]].

*S. alburnoides *is described as an allopolyploid hybridogenetic complex, where allopolyploid refers to an increased ploidy level and hybrid genome composition of particular forms within the system; hybridogenetic refers to an alternative mode of reproduction; and complex is the technical terminus denoting a natural system composed of parental species and their hybrids, with altered modes of reproduction and reproductive interdependence [10].

Presently, and due to the altered reproductive modes adopted by *S. alburnoides *and the reproductive relationship established with several allopatric bisexual *Squalius *species, mainly *S. carolitertii *(C genome) and *S. pyrenaicus*, a multitude of ploidy levels and genomic constitutions can be found [10]. These include diploids (PA, CA), triploids (PAA, PPA, CAA, CCA) and tetraploids (PPAA, CCAA) depending on the geographical location (Additional file 1, Figure S1). In the Iberian southern basins an additional form is present, composed exclusively of males designated as "nuclear non-hybrid AA's". These males are also considered hybrids because they carry mtDNA of *S. pyrenaicus *[6], despite their nuclear non-hybrid genome composition that is maintained through the reproductive dynamics of the complex [reviewed in [11]].

Being composed of allopolyploid individuals, the *S. alburnoides *complex is suited for qualitative and quantitative assessments of allele-specific transcriptional control (e.g. P and A). In a recent work, Pala *et al. *[12] showed a preferential expression of A alleles and an absence of P allele transcripts in most PAA triploids from one southern population (Sorraia River, Tejo basin, additional file 1, Figure S1). Contrastingly, in two analysed northern populations (from Douro and Mondego river basins), for the majority of individuals, both C and A genome alleles were simultaneously detected, irrespective of ploidy level or genomic composition. As such, the different patterns of allele usage found within the complex correlate with the presence of P or C genomes in the hybrid triploid forms, suggesting that differential expression regulation is due to differential genome interactions [12]. Nonetheless, while for C-containing forms the specimens were collected from two distinct Northern river basins, the P-containing individuals were all from the same river (Sorraia, Tejo basin) [12,13]. Thus, this phenomenon could not be considered to be generally connected to the simultaneous presence of P and A genomes, or whether it is a population-specific feature of the Sorraia River and/or Tejo basin. This, however, is crucial information to better understand the putative genomic interactions and/or other mechanisms regulating gene transcription dynamics in this allopolyploid organism.

The overrepresentation in whole organ extracts of a specific allele could be explained by the presence of several cell types, contributing unevenly to the total RNA extracted. Moreover, this effect can be more evident in an allopolyploid context when comparing organ-specific expression patterns between individuals of different ploidy and genomic constitutions. As such, the detection of expression differences between individuals and/or between organs can be the result of mosaicism within an organ and of different levels of mosaicism between organs. Indeed, ploidy mosaicism is well established and documented in vertebrates [14,15]. Natural ploidy mosaicism appears often associated with interspecific hybridization, as in the case of the reproductive complexes of the fish *Poecilia formosa *[16], *Cobitis taenia *[17] and lizards of the genus *Lacerta *[18]. Hence, in this context, the *S. alburnoides *complex is a likely candidate for the occurrence of this phenomenon. Moreover, in some species like *Platemys platycephala *diploid-triploid mosaics appear to be geographically and population dependent [19].

To determine ploidy and gene expression profiles, we developed a flow cytometry and cell sorting protocol for *S. alburnoides *tissues. This ensured a more homogeneous cells sampling for each organ with respect to cell number, size and complexity. In these samples we determined the expression profile of three widely expressed genes (*rpl8*, *gapdh *and *β-actin*) in liver and kidney of diploid and triploid forms of *S. alburnoides *from three major Portuguese southern river basins.

## Methods

### (a) Specimens collection, preliminary genotyping and preparation of cell suspensions

Samples of *S. alburnoides *and *S. pyrenaicus *were collected (and handled) with the approval of the portuguese National Forest Authority (AFN, fishing credential n° 29/2011) from several locations, distributed by three major river basins, corresponding to the southern distribution range of the complex in sympatry with *S. pyrenaicus *(Tejo, Guadiana and Almargem basins) (Additional file 1, Figure S1). All individuals were brought alive to the laboratory, morphologically identified and maintained under international ethical guidelines (ASAB, 2006).

From each individual a fin clip was obtained and each specimen was identified following the method described in Morgado-Santos *et al. *[20]. DNA was obtained by standard phenol/chloroform extraction from fins and the specimens were genotyped according to Inácio *et al. *[21]. Each individual was sacrificed with an overdose of the anaesthetic MS222 and blood was collected directly from the heart, diluted in freezing solution (40 mM citric acid trisodium salt, 0.25 M sucrose, and 5% dimethyl sulfoxide) and immediately frozen at -80°C for at least 30 minutes (to allow stabilization). Liver and kidney were collected and immediately digested for 15 minutes in 0.25% Trypsin (Sigma) and mechanically dissociated/homogenized using 26 G needle syringes. A HBSS solution containing 2% FBS was added to each sample to inactivate the enzymes and an 1100 rpm centrifugation for 8 minutes at 4°C was performed. Cells were resuspended in a HBSS + 2% FBS solution and filtered through a 40 μm nylon mesh. Cell numbers, morphology and viability (percentage of living cells from each organ after digestion treatment) were assessed using a Hemocytometer and Trypan blue staining.

### (b) Ploidy assessment

After preparation of the cell suspensions from liver, kidney and blood, nuclear staining was performed to assess ploidy diversity among cells of each organ in a subsample of each cell suspension. DRAQ5 (Biostatus) was added to aliquots of 0.5 × 10^6 ^or 1 × 10^6 ^cells of each cell suspension according to manufacturer instructions.

Chicken blood (2.5 pg of DNA per erythrocyte) was used as standard.

Cells were analysed on a FACSAria cytometer (BD Biosciences, San Jose, CA) equipped with both a 488 nm (15 mW output) Coherent Sapphire solid state laser (for light scatter analysis) and a 633 nm (18 mW output) JDS Uniphase HeNe air cooled laser for Draq5 excitation. Draq5 emission was detected using a 660/20 bandpass filter. Data was acquired using FACSDiva software (BD Biosciences, San Jose, CA) and acquisition of cells was performed with gating to exclude cell doublets and debris (FSC-W x FSC-A). The total number of collected events for ploidy determination was >10,000 per sample.

### (c) Cell sorting

To the remaining fraction of the cell suspensions of liver and kidney (DRAQ5 free), propidium iodide (P.I.: 1/5 of stock solution at 0.5 ng/ml) was added and incubated for 20 min at room temperature. Cells were analysed on a FACSAria high-speed cell sorter using the 488 nm (15 mW output) Coherent Sapphire solid state laser for light scatter analysis and P.I. excitation. P.I. emission was detected using a 695/40 band-pass filter. Data were acquired using FACSDiva software and acquisition of cells was performed with gating to exclude cell doublets and debris (FSC-W x FSC-A), and dead cells (P.I. positive).

From the light scatter dot plots (FSC-A x SSC-A) obtained from each organ digestion, a consistent pattern of events was identified between samples of the same organ, and two main regions (A and B: A_L _and B_L _in liver, A_K _and B_K _in kidney) were defined for each organ. For a set of individuals that presented homogeneous ploidy level, one region (B_L _from liver and B_K _from kidney) was chosen for cell sorting to increase the intra and inter sample homogeneity. Also, from three non-mosaic individuals (Sq18, Sq29 and Sq31), composed exclusively of 3 n cells, both A and B populations from both organs were sorted to assess whether expression mosaics correlate with different cell types. In one of the individuals where ploidy mosaicism was detected, both regions (A and B) from each organ were independently sorted because they roughly corresponded to 2 n and 3 n cells.

At least 2 replicates of 100,000 cells were sorted from each organ/fish directly to Buffer RLT Plus of the AllPrep DNA/RNA Mini Kit (Qiagen) and immediately frozen at -80°C for posterior nucleic acid extraction.

### (d) Genotyping and genome expression determination of the sorted cells

RNA and DNA were obtained from the previously frozen cells using AllPrep DNA/RNA Mini Kit (Qiagen).

The isolated DNA of B_L _sorted cell population of each fish was used as template for the amplification of *ß-actin *gene. Genotyping of that cell population was performed based on analyses of *ß-actin *PCR products according to Sousa-Santos *et al. *[22].

From the extracted RNA, first-strand cDNA was synthesized with RevertAid First Strand cDNA Synthesis Kit (Fermentas) by using oligo dT primers. Three genes, *ß-actin*, *rpl8 *and *gapdh *were amplified with specific primers (Additional file 2, Table S1) and according to the following PCR conditions: pre-heating at 94°C for 5 min, 35 cycles at 94°C for 1 min, 53°C (*rpl8*)/56°C (*gapdh *and *ß-actin*) for 1 min and 72°C for 1 min 30 s and a final extension at 72°C for 15 min. The PCR products were directly sequenced and analysed. Polymorphic sites for the two genomes (P and A) for Almargem and Guadiana fish populations were identified for the three genes using genome control sequences obtained from *S. pyrenaicus *and "nuclear non-hybrid" *S. alburnoides *from the mentioned rivers [GenBank accession numbers: JN790945; JN802520-JN802528; JN813376-JN802582]. For Tejo specimens the work of Pala *et al. *[12,13] provided the sequences for Tejo P and A genome specific polymorphisms for the three genes [EU199435-6; EU542913-6]. In hybrid samples, the presence of cDNAs derived from single genome copies or from both genomes was determined through sequence comparison by sequence alignment using Sequencher ver. 4.0 (Gene Codes Corporation, Inc.) and based on the identified polymorphic sites between genomes (P and A). Forward and reverse sequences for each gene were obtained per individual/per organ.

## Results

### (a) Intra-organ differences in ploidy - Detection of mosaic individuals

A total of 40 fish were analysed using flow cytometry for ploidy determination in blood, liver and kidney cell suspensions: four *S. pyrenaicus*, three nuclear non-hybrid *S. alburnoides *and 33 hybrids *S. alburnoides *(Table 1).

**Table 1:** Specimens' genotype, river basin, stream of capture and ploidy status in liver, kidney and blood

**Genotype**^**1**^	Code	Basin	Stream	Liver		Kidney		Blood
				**A**_**L**_	**B**_**L**_	**A**_**K**_	**B**_**K**_	**A**_**B**_
AA	Sq1	Almargem	Almargem	2n	2n	2n	2n	2n

	Sq22;							
AA	Sq23	Guadiana	Murtega	2n	2n	2n	2n	2n

PA	Sq6^2^	Almargem	Almargem	3n	2n	2n/3n	3n	2n

PA	Sq7; Sq8	Almargem	Almargem	2n	2n	2n	2n	2n

	Sq24;							
PA	Sq25;	Guadiana	Foupana	2n	2n	2n	2n	2n
	Sq26							

PA	Sq27	Guadiana	Murtega	2n	2n	2n	2n	2n

PA	Sq32	Tejo	Ocreza	2n	2n	2n	2n	2n

	Sq12;							
	Sq13;							
	Sq14;							
	Sq15;							
PAA	Sq17;	Almargem	Almargem	3n	3n	3n	3n	3n
	Sq18;							
	Sq19;							
	Sq20;							
	Sq21							

PAA	Sq11^2^	Almargem	Almargem	3n	2n	2n/3n	3n	3n

PAA	Sq16^2^	Almargem	Almargem	3n	2n	3n	2n	2n/3n

PAA	Sq28;	Guadiana	Murtega	3n	3n	3n	3n	3n
	Sq29							

PAA	Sq30^2^	Guadiana	Caia	3n	2n	3n	2n	3n

PAA	Sq31	Guadiana	Caia	3n	3n	3n	3n	3n

	Sq33;							
PAA	Sq34;	Tejo	Ocreza	3n	3n	3n	3n	3n
	Sq35							

	Sq39;							
PAA	Sq40	Tejo	Sorraia	3n	3n	3n	3n	3n

PP	Sq2; Sq3;	Almargem	Almargem	2n	2n	2n	2n	2n
	Sq4; Sq5							

	Sq36;							
PPA	Sq37	Tejo	Ocreza	3n	3n	3n	3n	3n

PPA	Sq38	Tejo	Sorraia	3n	3n	3n	3n	3n

^1^Genotyping from DNA extracted from fin clips

^2^Ploidy mosaic specimen

A_L _and B_L _defined cell dot regions in liver; A_K _and B_K _cell dot regions in kidney

**Figure 1:**
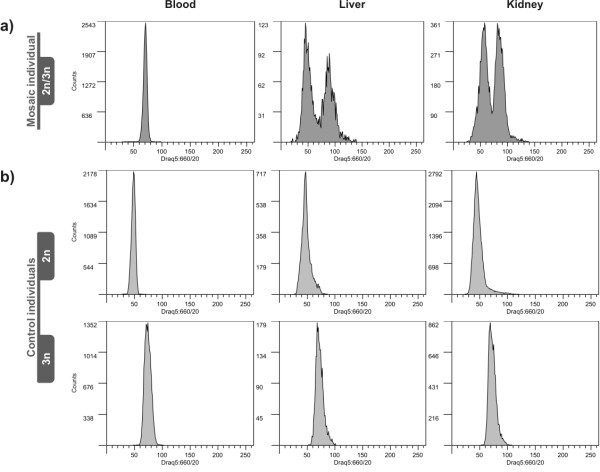
**DNA flow histograms of *S. alburnoides *liver, kidney and whole blood cell suspensions**. (a) example of one 2n/3n mosaic specimen, (b) control diploid and triploid specimens. Plots obtained from the analysis of Sq30; Sq2 and Sq9 respectively.

All the analysed *S. pyrenaicus *and nuclear non-hybrids *S. alburnoides *displayed exclusively diploid cells in liver, kidney and blood. From the analysis of the hybrid individuals, four were identified as ploidy mosaics (Figure 1a; Table 1): three from Almargem and one from Guadiana. In all four specimens, mosaicism was detected both in liver and in kidney but not in blood (Figure 1a). For mosaic individuals, the percentage of 2n and 3n cells within each organ was assessed (Table 2). In kidney, the percentages of 2n and 3n cells were quite constant between individuals, amounting to around 50%. In liver, the inter-individual variability was higher, and in three of the four cases there were more of 3n than 2n cells composing the organ. In blood, 100% of the cells were diploid in one mosaic specimen and 100% triploid in two others. In the fourth mosaic specimen vestigial amounts, less than 1.5%, of 2n cells were detected.

**Table 2:** Percentage of diploid and triploid cells in liver, kidney and blood of mosaic *S. alburnoides*

Code	Liver cells		Kidney cells		Blood	
	2n (%)	3n (%)	2n (%)	3n (%)	2n (%)	3n (%)
Sq6	31	69	56,6	43,4	100	0
Sq11	23,6	76,4	54,9	45,1	0	100
Sq16	20,6	79,4	52	48	1,3	98,7
Sq30	58,5	41,5	51,2	48,8	0	100

### (b) Determination of genotype and allele-specific expression in mosaic and non-mosaic individuals

**Figure 2:**
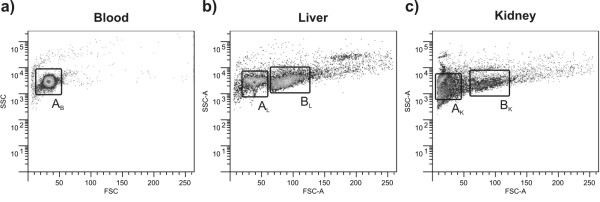
**Typical FSC_vs_SSC dot plots obtained from the tissues digestion**. (a) whole blood (b) liver and (c) kidney cell suspensions of *S. alburnoides*. Plots obtained from the analysis of Sq30.

From the analysis of each cell suspension in the flow cytometer a light scatter dot plot (FSC-A x SSC-A) of each organ was obtained for all individuals (Figure 2). The light scatter dot plots from all blood samples presented just one homogenous population and one region was detected (A_B_) (Figure 2a). From the light scatter plots obtained from liver and kidney, despite some variability found between individuals, two main dot regions, (A and B: A_L _and B_L _in liver, A_K _and B_K _in kidney) could be identified for each organ for each specimen (Figure 2b and 2c).

#### b1) Gene expression patterns according to organ and geographical location

The allele expression pattern of *ß*-*actin*, *rpl8 *and *gapdh *genes of B_K _and B_L _cells was assessed for a total of 20 individuals pooled from the Tejo, Almargem and Guadiana samples (Table 3). As expected, all PA individuals, regardless of the basin of origin, expressed simultaneously A and P alleles (biallelic expression) in both analysed organs for the 3 analysed genes (*ß*-*actin*, *rpl8 *and *gapdh*). In triploid PAA's from Guadiana, the expression of all 3 genes was also biallelic, both in liver and kidney. *rpl8 *expression was as well consistently biallelic in both organs in all analysed triploid PAA's. On the other hand, the expression profile of *ß*-*actin *and *gapdh *in PAA individuals from Almargem and Tejo was more variable. Despite the majority of biallelic expression detected for the 3 genes in both organs in the individuals from Almargem, there was one individual (Sq9) where only A-*gapdh *genome transcripts were detected in kidney and in liver samples. In two individuals from Tejo (Sq33 and Sq40), only A allele expression of *gapdh *was detected in kidney, but it was biallelic in the liver of these specimens and in both organs of the other Tejo individuals. *ß*-*actin *expression was biallelic in liver and kidney of all individuals irrespective of the geographic origin, except in the liver of one Almargem specimen (Sq14), which presented only A transcripts.

**Table 3:** β*-actin, rpl8 *and *gapdh *P and A allele-specific transcripts detected in liver and kidney cells of individuals from Almargem, Guadiana and Tejo populations of the *S. alburnoides *complex

Code	River Basin	River site	Ploidy	**Genotype**^**1**^	Liver expression			Kidney expression		
					**β*-actin***	***rpl8***	***gapdh***	**β*-actin***	***rpl8***	***gapdh***

Sq22	Guadiana	Murtega	2n	AA	A	A	A	A	A	A
Sq23	Guadiana	Murtega	2n	AA	A	A	A	A	A	A
Sq1	Almargem	Almargem	2n	AA	A	A	A	A	A	A
Sq3	Almargem	Almargem	2n	PP	P	P	P	P	P	P
Sq4	Almargem	Almargem	2n	PP	P	P	P	P	P	P
Sq5	Almargem	Almargem	2n	PP	P	P	P	P	P	P
Sq27	Guadiana	Murtega	2n	PA	PA	PA	PA	PA	PA	PA
Sq8	Almargem	Almargem	2n	PA	PA	PA	PA	PA	PA	PA
Sq29	Guadiana	Foupana	3n/3n	PAA	PA/PA	PA/PA	PA/PA	PA/PA	PA/PA	PA/PA
Sq31	Guadiana	Caia	3n/3n	PAA	PA/PA	PA/PA	PA/PA	PA/PA	PA/PA	PA/PA
Sq9	Almargem	Almargem	3n	PAA	PA	PA	A	PA	PA	A
Sq13	Almargem	Almargem	3n	PAA	PA	PA	PA	PA	PA	PA
Sq14	Almargem	Almargem	3n	PAA	A	PA	PA	PA	PA	PA
Sq18	Almargem	Almargem	3n/3n	PAA	PA/PA	PA/PA	PA/PA	PA/PA	PA/PA	PA/PA
Sq16	Almargem	Almargem	3n/2n	PAA/AA	PA/A	PA/A	PA/A	PA/A	PA/A	PA/A
Sq38	Tejo	Sorraia	3n	PPA	PA	PA	PA	PA	PA	PA
Sq40	Tejo	Sorraia	3n	PAA	PA	PA	PA	PA	PA	A
Sq36	Tejo	Ocreza	3n	PPA	PA	PA	PA	PA	PA	PA
Sq37	Tejo	Ocreza	3n	PPA	PA	PA	PA	PA	PA	PA
Sq33	Tejo	Ocreza	3n	PAA	PA	PA	PA	PA	PA	A

^1^DNA extracted from liver cells

The expression pattern of triploid PPA's from Tejo was also determined for *ß*-*actin*, *rpl8 *and *gapdh *genes, and it was found to be biallelic for the 3 genes in both organs.

The genotype of both A and B cells in liver and kidney of controls for expression mosaic (non ploidy mosaic triploid Sq18, Sq29 and Sq31) was PAA, and the expression outcome was biallelic (Table 3) for all the individuals for both organs and for both A and B cell fractions.

#### b2) Analysis of ploidy mosaics

In two of the individuals (Sq16 and Sq30) where ploidy mosaicism was detected, the 2n and 3n cell pools (P_3n _and P_2n_) in liver and kidney corresponded to the light scatter defined A and B regions in each organ (P_3n _= A_K _= A_L _and P_2n _= B_K _= B_L_) for both organs. This natural separation allowed sorting of 2n and 3n cells from liver and kidney without nuclear staining. The use of intercalating dyes for cellular DNA content measurements proved to be not compatible with on column DNA/RNA extraction (tested on samples Sq6 and Sq11, that were this way lost, data not shown). Only from Sq16 and Sq30 individual P_2n _and P_3n _sorted cells were isolated without nuclear staining but only from Sq16 good quality DNA and RNA were obtained from both diploid and triploid cell pools.

The 2n and 3n cell pools were genotyped as P_3n _= A_K _= A_L _= PAA genotype and P_2n _= B_K _= B_L _= AA genotype.

The genome specific allele expression of *gapdh, β-actin *and *rpl8 *in both 2n and 3n cell pools was as well assessed. It revealed that P_3n _= A_K _= A_L _where P and A transcripts were detected, and in P_2n _= B_K _= B_L _where only A transcripts were detected.

## Discussion

In the present work we studied the expression pattern of *S. alburnoides *specimens from three southern Portuguese drainages (Tejo, Guadiana and Almargem), using RNA obtained from homogeneous pools of cells form whole organs. We used flow cytometry and cell sorting to obtain homogeneous cell pools for RNA extraction and to screen for the occurrence of somatic ploidy mosaics in *S. alburnoides*.

Flow cytometry clearly revealed the occurrence of diploid-triploid mosaicism in *S. alburnoides *complex. The detected frequency of this phenomenon was approximately 10%, indicating that the diploid-triploid mosaics represent a non-regular component of the genetic system of this complex rather than a stably incorporated feature of its reproductive dynamics, as reported in *Platemys platycephala *[19] and *Liolaemus chiliensis *[23]. Interestingly, the observed 10% ploidy variation is qualitatively different from previous reports of the same nature such as *P. formosa *[16] where this frequency was 2 orders of magnitude lower. In this case, being the occurrence of mosaic *P. formosa *very rare, the phenomenon has been considered as a mistake of a complicated reproductive system without evolutionary meaning. On another hand, being higher, the *S. alburnoides *mosaic frequency raises questions about whether the phenomenon has a real impact on the evolutionary dynamics of the species.

According to Dawley and Goddard [14], there are two possible main mechanisms that lead to diploid/triploid mosaicism: delayed fertilization and genome loss. Delayed fertilization occurs when the sperm pronucleus is slow to fuse with the female pronucleus and so, fails to participate in the first mitotic division. In this case the sperm nucleus is kept in one of the daughter cells (blastomeres) and fuses with a maternal nucleus only later, after a variable number of mitotic divisions. Consequently, a mosaic arises with triploid cells resulting from fertilization and diploid cells resulting from an initial "gynogenetic" development. This is the case of the diploid-triploid mosaics of *Misgurnus anguillicaudatus *[24] and possibly of the naturally occurring 2n/3n mosaic *P. formosa *[16]. Another mechanism is genome loss. Here, one parental chromosome set is selectively eliminated. This selective loss of a whole genomic set has been documented to occur during oogenesis of hybridogenetic unisexuals, such as *Rana esculenta *[25] and *Bufo pseudoraddei baturae *[26]. Both of the above mentioned mechanisms may be causing mosaicism in *S. alburnoides*, since this hybrid complex presents many reproductive pathways with altered oogenesis (with genomic exclusion) and spermatogenesis [reviewed in [11]].

**Figure 3:**
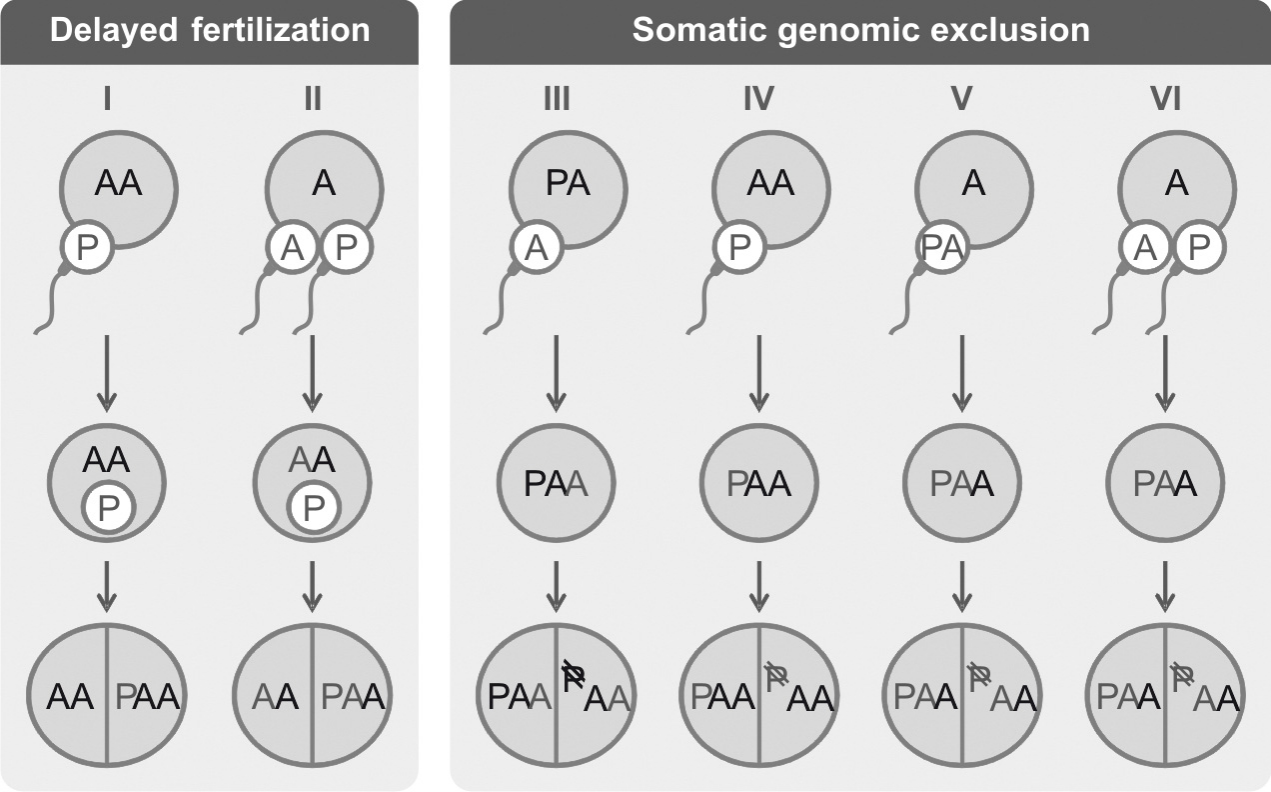
**Possible mechanisms leading to the formation of 2n(AA)/3n(PAA) mosaic *S. alburnoides***. Alternative developmental routs within the two main mechanisms of mosaic establishment (delayed fertilization and genomic exclusion) that can lead to the formation of 2n(AA)/3n(PAA) mosaics. Eggs (large circles) and sperm (small circles with tail) contribute with P and A genomic complements (maternal genomic contributions in black and paternal genomic contributions in grey).

Considering the 2n(AA)/3n(PAA) mosaic individual (Sq16), the possible routes (Figure 3) leading to this mixed genotype can be explained considering the reproductive modes of the different *S. alburnoides *forms [reviewed in [11]]. PAA triploid individuals are the most abundant form of the complex, and they are normally produced throughout the syngamy of a diploid PA oocyte with a haploid A sperm, or also by syngamy of one haploid A oocyte with a diploid PA sperm. Although uncommon, other path that leads to PAA formation is the syngamy of a diploid AA oocyte (produced by PAA females) with a haploid P sperm. If a P sperm nucleus enters a diploid AA ovum, initially remaining quiescent but later undergoing amphimixis with an early cleavage cell (Figure 3, route I), a 2n(AA)/3n(PAA) mosaic individual would arise through delayed fertilization. Another delayed fertilization scenario that could lead to the occurrence of a 2n(AA)/3n(PAA) mosaic is dispermy (Figure 3, route II). In such case a haploid oocyte has been fertilized by two sperm cells carrying distinct genomics sets. If karyogamy occurred only between the oocyte nucleus (A) and the sperm carrying the homologous genome, while the P sperm nucleus remains inactive during one or more embryo cleavages and only later fusing with an AA blastomere, a chimeric 2n(AA)/3n(PAA) organism would be obtained.

The 2n(AA)/3n(PAA) mosaics may also result from the loss of a whole P genome from single dividing cells in a triploid PAA embryo (Figure 3 routs III; IV, V and VI). Genomic exclusion is documented to occur during gametogenesis in hybridogenetic unisexuals, *S. alburnoides *including. Studies in the hybridogenetic water frogs *Pelophylax esculentus *[27] revealed that the genome exclusion from the germ line occurs prior to meiosis, during the prolonged phase of oogonial proliferation. So, the extension of this phenomenon to non-germinal lineages is not a big leap. In fact, the process of elimination of chromatin from pre-somatic and somatic cells is not an oddity, being in fact a very common mechanism in differentiation and development [reviewed in [3]]. The viable occurrence of 2n/3n human mosaics (or mixoploids) is also known [reviewed in [28]] and was, at least circumstantially, related to genomic exclusion and a phenomenon described as postzygotic diploidization. These human mixoploids had two paternal genomic contributions, so they originated through a process similar of what is illustrated in routes V or VI of Figure 3.

Regarding the other *S. alburnoides *specimens diagnosed as 2n/3n mosaics, we were not able to genotype the 2n and 3n cell populations from liver and kidney, so they might present other genomic compositions than 2n(AA)/3n(PAA). Therefore, the possible ways and routes that could lead to *S. alburnoides *2n/3n mosaics may go beyond the ones sketched in Figure 3.

Another aspect worth discussing is the percentage of triploid and diploid cells that characterizes the mosaic individuals. According to Lamatsch *et al. *[16], either in the mosaics resulting from delayed fertilization or from genomic exclusion (if occurring early in development), a greater proportion of diploid cells, compared to triploid ones, would be expected. Occurring early in development, due to the lower DNA content, these diploid cells should probably replicate their DNA faster than the triploid cells and would, therefore, be able to divide more often than triploid cells. Nilsson and Cloud [29] postulated that in organs in which cells are rapidly replicating, triploid cells are prone to lose extra chromosomes and resume diploidy. So, if our results point to a phenomenon of postzygotic diploidization by genomic exclusion, it occurred in a not so early stage of development, since no strong bias was detected towards diploid cells (Table 2).

An unexpected result was found in blood ploidy measures. In this tissue, 100% of the cells were triploid in two of the mosaic specimens, 100% diploid in another and some vestigial 2n cells were detected in one sample (less than 1.5%). Some cases confirm that the use of blood is an accurate determinate of overall ploidy levels [19], once the comparison of the proportions of diploid and triploid cells in the blood with the ones determined in other tissues of the same individual, it showed only minor deviation. On the contrary, in our case, if only blood have been analysed, the mosaics would have been misdiagnosed as complete diploid and/or triploid individuals. The reasons why mosaicism is not present in the *S. alburnoides *blood samples is difficult to explain, but also in some specimens of the mosaic *P. platycephala*, blood presented a non-mosaic phenotype while some solid tissues of that same specimens were clearly 2n/3n mixoploid [19]. In one case reported in humans, a 46,XX/69,XXY mosaic also displayed a similar variation between tissues. While the 2n/3n ratio was 2:3 in fibroblasts, in blood (lymphocytes) the ratio was 24:1 [30]. An explanation for these results is that the blood is derived from the hematopoetic stem cells and has a continuous proliferating ancestry which is different to kidney and liver. While kidney and liver mosaicism may reflect a situation that goes back to the embryo when both organs were formed, the blood is reflecting the adult situation. It may well be that in the hematopoetic stem cell pool only one type of the two ploidy stages will become more prevalent. If one ploidy state is advantageous, there might be selection in the multiple rounds of hematopoetic stem cell divisions. So 2n could be faster cycling than 3n and finally only 2n cells will be seen. On the other hand 3n stem cells might have a greater allelic repertoire and this could be advantageous.

The choice of liver, kidneys and blood as target organs was related to technical issues, because the procedure was attempted also in other organs but with no success. The analysis of gonads would have been particularly interesting because it is know from experimental crosses that triploid *S. alburnoides *females can in fact, sporadically produce haploid and triploid eggs [7].

Beyond the existence of ploidy mosaicism, also the possible occurrence of expression mosaics within the organs was cursorily prospected (Table 1: Sq18, Sq29 and Sq31). No differences were detected, neither between cell populations nor genes, being the expression pattern constantly biallelic (PA) so we have not found expression mosaicism at this level of analysis.

The prospection for mosaicism was one of the goals of this work because if happening it could have some impact in the expression patterns within and between organs. The pattern of preferential homologue genome usage previously detected for Tejo (Sorraia River population) [12,13] could have been affected or biased due to mosaicism. So, we analysed the expression pattern of three genes, *rpl8*, *gapdh *and *β-actin*, for several *S. alburnoides *individuals (which ploidy status had been assessed), not only from Sorraia River (Tejo basin), but also from some other populations of Tejo and other southern drainages (Guadiana and Almargem). We detected for all analysed specimens from Tejo a preferential biallelic expression in the cells sorted, both from liver and kidney, for *β-actin *and *rpl8 *genes, and also in liver cells for *gapdh *gene. Nevertheless, P genome transcripts of *gapdh *were not detected in the kidney cells of two non-mosaic triploid PAA's, one coming from Sorraia and one from Ocreza. Consequently, we can conclude that a) the detection of only A transcripts is a phenomenon independent of ploidy mosaicism; and b) although P genomic complement is present, it is not transcribed in some tissues and from some genes, as presented and discussed by Pala *et al. *[13]. This allele silencing is not restricted to individuals from a single river (Sorraia), but also occurs in other river (Ocreza) from the same drainage (Tejo basin), and in different drainages (observed also in Almargem basin), along the range of sympatry with *S. pyrenaicus*.

When a preferential allelic usage of A in PAA fish happens, that could be interpreted as a matter of genomic homology. If genomic homology plays a role in regulating allelic expression we would predict that in PPA individuals we should detect P expression, predominantly. Therefore, we extended the analysis of Tejo triploid *S. alburnoides *to three PPA individuals, a previously not analysed genomic constitution. For these animals, expression is constantly biallelic (PA) suggesting that genetic homology is unlikely to be at play in regulating the profiles of allelic expression of triploid individuals.

Also, the occasional occurrence of ploidy mosaics does not correlate with the sporadically absent P allele expression. Only A allele expression was observed to occur in non-mosaic individuals, and when analysing the expression pattern of the Sq16 mosaic specimen (2n-AA/3n-PAA), the expression was biallelic (PA) for the 3n (PAA) cells despite the monoallelic (A) expression of the 2n (AA) cells that composed the organs of that individual.

In this work we detected the occurrence of ploidy mosaics among *S. alburnoides *specimens, but we could discard the influence of this phenomenon on the detection of variable allelic expression profiles in triploid individuals. Alternatively, as previously proposed [13], the absence of P allele transcripts in some genes of triploid PAA *S. alburnoides*, as we also report (Table 3), can be explained by the occurrence of compensation by gene-copy silencing. Consequently, PAA' triploid individuals would only transcribe two alleles per gene (PA or AA or PA'). In fact, some studies predominantly in polyploid plants [31,32] have been pointing to a process of functional diploidization as a way to balance gene dosage [33]. So, if a functional diploidization is necessary and is in fact the way through which *S. alburnoides *can cope with allopolyploidy, the ploidy status of the organism is not relevant. In this scenario, the occurrence of mixoploidy may emerge from the relaxation of the mechanisms that impose a tight control over mitosis and ploidy control.

## Conclusions

We have shown that ploidy mosaicism occurs sporadically within the *S. alburnoides *complex, but in a frequency significantly higher than reported for other organisms. Moreover, we could exclude the influence of this phenomenon on the detection of variable allelic expression profiles of ubiquitously expressed genes in cells from liver and kidney of triploid individuals.

Finally, we determined that the expression patterns previously detected only in a narrow geographic range is not a local restricted phenomenon but is widespread in rivers where *S. pyrenaicus *is sympatric with *S. alburnoides*.

Altogether, our results point to interesting avenues of research on the evolutionary and mechanistic interplay between mitotic checkpoints, polyploidization and mosaicism.

## Authors' contributions

MMC conceived and coordinated the study. IM was involved developing the work in all steps of the study and drafted the manuscript. ES and MS participated in the design of the study and in the critical revision of the manuscript. MPM participated in cytometry and cell sorting. RG supervised the cytometry and cell sorting assays. AI participated in fish genotyping. All authors participated in the discussion of the results and read and approved the final manuscript.

## Supplementary Material

Additional file 1 **Figure S1-Distribution of *S. alburnoides *in Portugal and areas of sympatry with other *Squalius *species involved in the *S. alburnoides *polyploid reproductive complex**. Figure S1-Distribution of *S. alburnoides *in Portugal and areas of sympatry with other *Squalius *species involved in the *S. alburnoides *polyploidy reproductive complex. Distribution of *S. alburnoides *in Portugal and areas of sympatry with other *Squalius *species involved in the *S. alburnoides *polyploid reproductive complex. Rivers from which *S. alburnoides *and *S. pyrenaicus *were sampled are marked in red in the first panel: a) Ocreza; b) Sorraia, c) Caia; d) Murtega; e) Foupana and f) Almargem. In the second panel the major Portuguese river basins are identified. Click here for file

Additional file 2 **Table S1. Primer sequences and references for each gene**. Table S1. Primer sequences and references for each gene. Primer sequences and references for each gene amplified for this work.. Click here for file
