# Supplementary material for: Ploidy mosaicism and allele-specific gene expression differences in the allopolyploid Squalius alburnoides
Source: BMC Genet. 2011 Dec 5;12:101. doi: 10.1186/1471-2156-12-101 (PMC3276436; doi:10.1186/1471-2156-12-101)
Supplement: Additional file 1 — Figure S1-Distribution of S. alburnoides in Portugal and areas of sympatry with other Squalius species involved in the S. alburnoides polyploid reproductive complex. Figure S1-Distribution of S. alburnoides in Portugal and areas of sympatry with other Squalius species involved in the S. alburnoides polyploidy reproductive complex. Distribution of S. alburnoides in Portugal and areas of sympatry with other Squalius species involved in the S. alburnoides polyploid reproductive complex. Rivers from which S. alburnoides and S. pyrenaicus were sampled are marked in red in the first panel: a) Ocreza; b) Sorraia, c) Caia; d) Murtega; e) Foupana and f) Almargem. In the second panel the major Portuguese river basins are identified. [file 1471-2156-12-101-S1.PDF]

Figure S1- Distribution of *S. alburnoides* in Portugal and areas of sympatry with other *Squalius* species involved in the *S. alburnoides* polyploid reproductive complex.

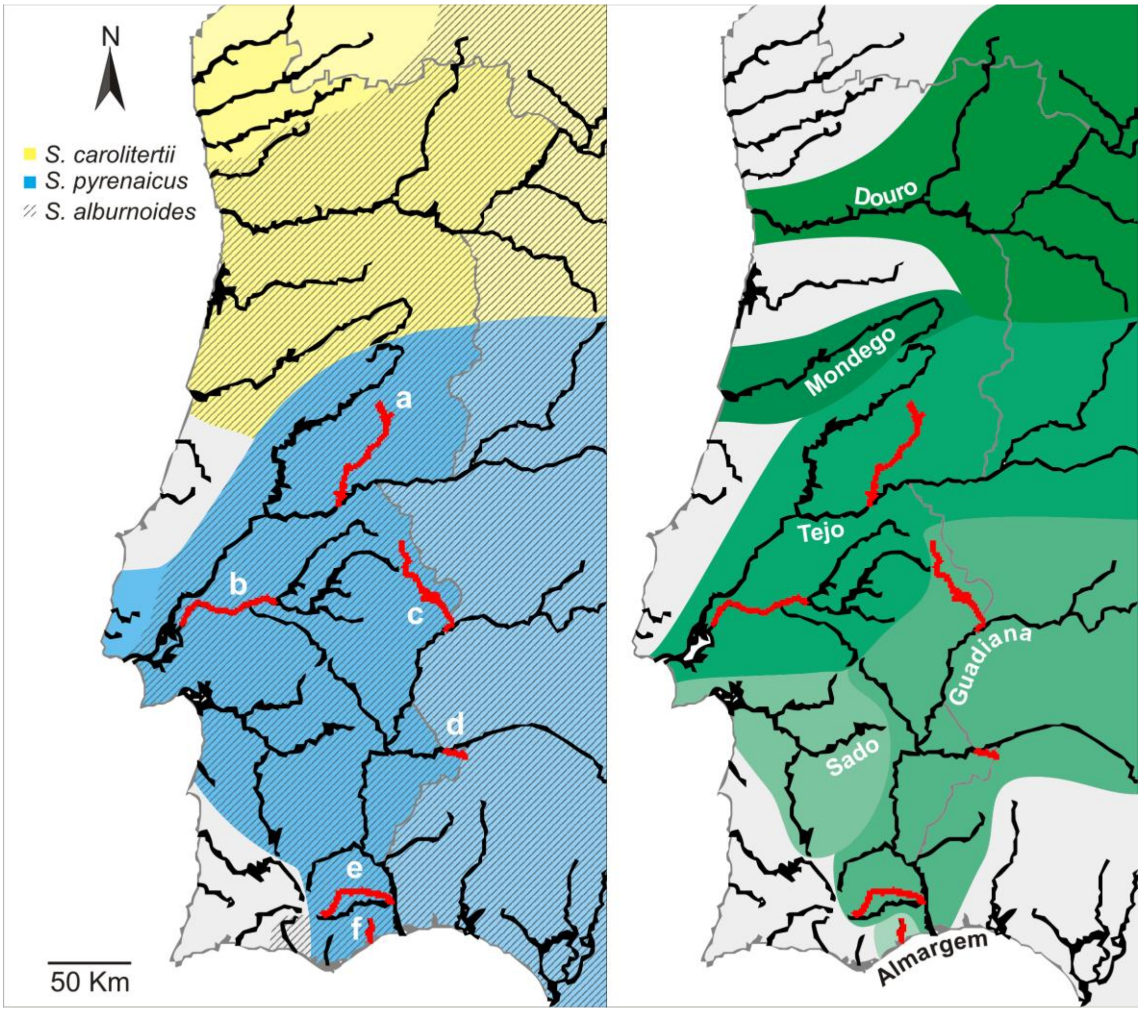

Rivers from which *S. alburnoides* and *S. pyrenaicus* were sampled are marked in red in the first panel: a) Ocreza; b) Sorraia, c) Caia; d) Murtega; e) Foupana and f) Almargem. In the second panel the major Portuguese river basins are identified.
