# Supplementary material for: Ploidy mosaicism and allele-specific gene expression differences in the allopolyploid Squalius alburnoides
Source: BMC Genet. 2011 Dec 5;12:101. doi: 10.1186/1471-2156-12-101 (PMC3276436; doi:10.1186/1471-2156-12-101)
Supplement: Additional file 2 — Table S1. Primer sequences and references for each gene. Table S1. Primer sequences and references for each gene. Primer sequences and references for each gene amplified for this work.. [file 1471-2156-12-101-S2.PDF]

Table S1. Primer sequences and references for each gene.

Gene	Primer	Sequence	References
ß-actin	ß-ACTIN-F1	5'-CAACGGCTCCGGCATGTG-3'	Pala *et al.*, 2008
	ß-ACTIN-R1	5'-TGCCAGGGTACATGGTGG-3'	Pala *et al.*, 2008
rpl8	Rpl8 forward	5'-CTCCGTCTTCAAAGCCCATGT-3'	Pala *et al.*, 2008
	Rpl8 reverse	5'-TGTTCCTCGCAGTCTGCCAG-3'	Pala *et al.*, 2008
gapdh	GAPDH-F1	5'-ATCAGGCATAATGGTTAAAGTTGG-3'	Pala *et al.*, 2008
	GAPDH-Ri	5'-GGCTGGGATAATGTTCTGAC-3'	-
